# Identification of *Lycopene epsilon cyclase* (*LCYE*) gene mutants to potentially increase β-carotene content in durum wheat (*Triticum turgidum* L.ssp. *durum*) through TILLING

**DOI:** 10.1371/journal.pone.0208948

**Published:** 2018-12-10

**Authors:** Daniela Richaud, Claudia Stange, Agata Gadaleta, Pasqualina Colasuonno, Roberto Parada, Andrés R. Schwember

**Affiliations:** 1 Laboratorio de Fitomejoramiento Molecular, Facultad de Agronomía e Ingeniería Forestal, Pontificia Universidad Católica de Chile, Santiago, Chile; 2 Laboratorio de Biología Molecular Vegetal, Facultad de Ciencias, Universidad de Chile, Santiago, Chile; 3 Department of Environmental and Territorial Sciences (DiSAAT), University of Bari “Aldo Moro”, Bari, Italy; Institute of Genetics and Developmental Biology Chinese Academy of Sciences, CHINA

## Abstract

Increasing β-carotene (a vitamin A precursor) content in *Triticum turgidum* L. ssp. *durum* (durum wheat) grains is important to improve pasta nutritional quality. Studies in other species show that altering the expression of *LCYE* genes increases the flux towards the β-β branch, accumulating higher β-carotene levels. Durum wheat is a tetraploid species that has two *LCYE* genes (*LCYE-A* and *LCYE-B*) associated to the A and B genomes. The objective of this work was to produce durum wheat *LCYE* mutants through EMS to potentially increase β-carotene content. The *LCYE* point mutations created with EMS were identified using a Kronos TILLING (Targeting Induced Local Lesion IN Genomes) mutant population. Specific primers that amplified exons 3 through 10 of the *LCYE* genes were designed and validated. To simplify the TILLING procedure, fragments were digested with CJE (Celery Juice Extract) and visualized on 2% agarose gels. 6X mutant pools were identified, which showed cleavage products and then made into 2X pools to identify mutant individuals. *LCYE* mutants were then sequenced and evaluated with BLOSUM62, SIFT and PSSM algorithms. Mutants with substitutions W437*, P334L and G368R in *LCYE-A* and P405L, G352R and T393I in *LCYE-B* predicted to affect protein function were selected. Substitution W437* increased β-carotene in 75% and overall total carotenoids content in leaves of the mutant 2426 (A1 mutant line), but no significant differences relative to the control were found in grains through HPLC. Finally, the increased levels of β-carotene on leaves have potential applications to improving plant resistance under contaminated environmental conditions.

## Introduction

World wheat production is currently around 728 millions tons in 2018 (http://www.fao.org/worldfoodsituation/csdb/en/), being one of the most important staple foods in the human diet, providing around 19% of the carbohydrates and 20% of proteins [1] by direct consumption. Durum wheat (*Triticum turgidum* L. ssp. *durum*) is a tetraploid species that comprises the A and the B genomes. It is the main source of semolina for pasta production, couscous and burghul, among others [2]. The yellow-amber color of semolina is one of the most important quality traits for durum wheat end- products, which is due to the yellow pigment content (YPC) in grains. YPC is composed by 30% - 50% of carotenoids [3, 4], pigments with important nutritional and health roles. There are two kinds of carotenoids; xanthophylls and carotenes, all of them have antioxidant properties that reduce the risk of chronic degenerative diseases [5, 6]. β-carotenes, α-carotenes and β-cryptoxanthin also have provitamin A activity, which provides protection from ocular diseases [7]. *Trans*-*β*-carotene is the most suitable and important precursor for vitamin A synthesis [8, 9] as β-carotene has two pro-vitamin A structures, whereas α-carotene and β-cryptoxanthin have only one [10]. Vitamin A deficiencies are a global health problem in undeveloped countries (i.e., Southeastern Asia), where it generates permanent blindness and increases sensitivity to infectious diseases [11]. The World Health Organization (WHO) estimates that 250 million pre-school children are deficient in vitamin A (retinol concentration <0.70 mmol/l), and 250,000 to 500,000 children become blind each year, and half of them die from accidents associated to sight loss (http://www.who.int/nutrition/topics/vad/en/).

The main carotenoid in mature durum wheat grains is lutein, a xanthophyll compound, which accounts for 86–94% of total carotenoids that corresponds to 2–5 μg/gdw [12]. Lutein is not essential in human diet, as there are no clinical conditions specifically related with lutein deficiency, so it has no known nutritional value to date [13]. Other carotenoids are present in smaller amounts, and β-carotene accumulation reaches only 0,15 μg/gdw [5, 12]. Large accumulation of β-carotene is an important trait in breeding programs [14] aimed at improving the nutritional quality of durum wheat grain and its final products. Carotenoid biosynthesis pathway has at least three rate limiting steps: (1) the condensation of two GeranylGeranyl diPhosphate (GGPP) molecules to produce phytoene by Phytoene Synthase (PSY), (2) lycopene cyclization and (3) carotene hydroxylation [2, 15]. There are two enzymes involved in lycopene cyclization that diverts the carotenoid biosynthetic pathway into two metabolic branches, the ε-β branch controlled by LCYE (Lycopene Epsilon Cyclase) and LCYB (Lycopene Beta Cyclase), which eventually produces lutein. In addition, the β-β branch is only controlled by LCYB that converts lycopene into β-carotene [16–19]. Genes involved in carotenoid biosynthesis in tetraploid wheat exhibit expression patterns that reflect the pigments primary functions at light harvesting and as photoprotective compounds in photoactive tissues, thus higher expression is observed in leaves, compared to stems and roots [20, 21]. The overexpression of the *LCYB* gene or the knockdown in *LCYE* expression is sufficient to increse total carotenoid and β-carotene content in plants. In tomato plastids transformed with *LCYB*, there was an unexpected increase in the total carotenoid content, with predominant accumulation of β-carotene in the fruits and xanthophyll carotenoids in the leaves [22]. In carrot, the overexpression of *LCYB1* produced up to 2 fold of increse in β-carotene in leaves and the storage root without affecting the lutein levels [23]. In *Arabidopsis*, the *lut2* mutant is deficient in the LCYE enzyme and its leaves did not synthesize lutein, but showed high levels of β-carotene in leaves [24]. *LCYE* silenced tomato plants showed an increase in total carotenoids and β-carotene, whereas lutein content decreased [25]. The same result was reported with rapeseeds, in which the RNAi *LCYE* silenced lines exhibited reduced expression of *LCYE* and increased levels of total carotenoids, including β-carotene, zeaxanthin, violaxanthin and, unexpectedly, lutein content [26]. In potato, tubers that were subjected to specific silencing of the *LCYE* gene also resulted in increased levels of β-carotene (up to 14-fold), with two transgenic lines showing slight increases in lutein content (1.5–1.8 fold) [27]. Studies in maize mutants showed that variations in the *LCYE* gene altered the pathway of α-carotenes, and the *LCYE* polymorphisms explained 58% of the variation between the two branches, increasing up to three times the content of provitamin A compounds [28].

In the hexaploid common wheat variety Chinese Spring, *LCYE* and *PSY-A1* genes co-localized with a QTL related to the content of lutein in the wheat endosperm, and a sequence change in *LCYE* may result in increased activity relative to β-cyclase, resulting in increased lutein content [29]. Also, they obtained and cloned the *LCYE-A* (NCBI Genbank: EU649785, 4,652 bp), *LCYE-B* (NCBI Genbank: EU649786, 4,435 bp) and *LCYE-D* (NCBI Genebank: EU649787, 4,392 bp) gene sequences by genome walking. All genes display 10 exons and a similar gene structure, with the only major difference being the length of intron 1 in the 3A homeoform [29]. Taken this evidence into account and the final aim to increase β-carotene content in durum wheat, we propose to select *LCYE* mutants from a durum wheat mutant population. However, genetic improvement in wheat is a complex challenge. The principal problems are the length of its genome (16,000 Mb in hexaploid wheat), highly repetitive DNA content (83%) [30], and homoeologous genes that share between 93–96% of their sequences. The TILLING strategy allows the identification of point mutations created with mutagens such as EMS (ethyl methane sulphonate), which alkalizes guanines leading them to pair with thymines [31]. This technique is a powerful reverse genetics approach for wheat due to the high mutation densities that polyploids can tolerate 1/51 kb for 50% GC targets [32]. Also, there are higher probabilities of identifying nonsense mutations that combined in a final genotype may substitute the wild type genes. The strategy of TILLING has been applied successfully in other studies in mutagenized populations of bread and durum wheat for genes involved in agronomic and quality traits [32–38]. Fu et al., 2009 [36] confirmed that the gene *Yr36* present in wild wheat, conferred resistance to a broad spectrum of stripe rust races. This gene includes a kinase (WKS1) and a putative START lipid-binding domain. They selected six TILLING mutants with changes in conserved amino acids in WKS1 and five showed susceptible reactions similar to the susceptible UC1041 control line, suggesting that WKS1 is necessary for the resistance response. TILLING mutant populations are already available. For example, populations of common wheat (cv. Cadenza, 4,200 lines, cv. Paragon, 6,000 lines) and durum wheat (cv. Cham1, 4,200 lines) are available under the Wheat Genetic Improvement Network (http://www.wgin.org.uk/). Therefore, the main objective of this work was to identify possible knockdown or knockout *LCYE* mutant*s* using a durum wheat TILLING mutant population and associate the mutant lines with increased β-carotene content in the plant. The identification of *LCYE* mutants has great value because they will allow the creation of new alleles, creating genetic variability that can enrich breeding programs and can also be used in functional genomics research, helping to unravel how the carotenoid biosynthetic pathway specifically works in wheat.

## Materials and methods

### Plant material

The genomic DNA from 1,370 individuals from the durum wheat Kronos TILLING mutant population was provided by Jorge Dubcovsky from the University of California in Davis [32]. All mutant lines were developed using EMS as chemical mutagen at 0.7–0.75% concentration.

### Genome-specific primers design

**Fig 1:**
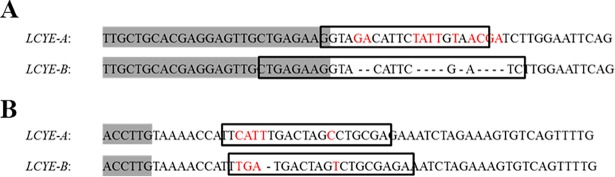
**Alignment of *LCYE-A* and *LCYE-B* sequences used to design genome-specific forward (A) and reverse (B) primers**. Primers are surrounded by boxes and genome-specific polymorphisms are indicated in red. Exon 3 (A) and exon 10 (B) are grey-highlighted and all the other sequences correspond to intronic regions. Indel events are depicted by dashed lines. The specific primers used listed from top to bottom are LCYE-AF1, LCYE-BF1, LCYE-AR1, and LCYE-BR1.

To amplify and sequence the *LCYE* gene, a specific region of 1,500–1,700 bp (Fragment 1, comprising exons 3 to 10 within the *LCYE* gene) was chosen. Regions with conserved exons, high GC content and high probability of containing missense or nonsense mutations (which code for a stop codon or mutations in splice junctions sites) were prioritized. This information was obtained from CODDLE (Choose Codons to Optimize the Detection of Deleterious LEsions, http://www.proweb.org/coddle) web software, which evaluates possible effects of relevant mutations in protein function. Primers were designed based on the sequence of the *LCYE* genes of the hexaploid variety Chinese Spring: *LCYE-A* (Genbank: EU649785) and *LCYE-B* (Genbank: EU649786). Genome specific primers were designed complementary to intronic regions that had INDELS and SNPs between homoeologous copies (Fig 1, Table 1) to amplify a 1,700 bp fragment (fragment 1).

**Table 1:** LCYE primers sequence and their chromosome localization.

Primer	Sequence (5´-3´)	Description
LCYE-AF1	GGTAGACATTCTATTGTAAC	Chromosome 3A specific
LCYE-AR1	TCGCAGGCTAGTCAAATGAA	
LCYE-AF2	CAGTGTGCTTCTGCAATGGA	
LCYE-AR2	ACCAAGCATTGATGGACTGG	
LCYE-BF1	CTGAGAAGGTACATTCGATC	Chromosome 3B specific
LCYE-BR1	TCTCGCAGACTAGTCATCAA	
LCYE-BF2	ACACACCCTGAGGAAGCCAA	Non-chromosome 3B specific
LCYE-BR2	GGATGGACCATACTCGCTGC	

For nested PCR, primers were designed within the *LCYE* fragment 1 to amplify fragment 1a and fragment 1b, whose lengths are approximately 1,200 bp each. The sequences for *LCYE-A* and *LCYE-B* and the Primer3 v.4.0 software [39] were used to design these primers (Table 1).

### Genome-specific primers validation

To assure that primers were genome specific, they were validated using Langdon D-genome disomic substitutions lines LDN-3D(3A) and LDN-3D(3B) obtained from the United States Department of Agriculture (USDA) (S1 Fig). In these lines, the A and the B genomes are substituted by the D genome in LDN-3D(3A) and LDN-3D(3B), respectively.

### Identification of mutant pools

This protocol is based on the two-step screening approach described by Uauy et al., 2009 [32]. First, the same amount of DNA from each individual from the Kronos TILLING mutants population (i.e., 1,370 individuals) were mixed into a six fold pool (6X pools, meaning that 6 individuals named as A, B, C, D, E and F of that population were mixed) and organized into 96-well plates. Every 6X pool was given a correlative code from 1 through 228 and 25 μL touchdown PCR reactions were performed (S1 Appendix and S1 Table). A denaturing and re-annealing step is included at the end of the PCR reaction (99°C for 10 min, 70 cycles of 70°C for 20 s decreasing 0.3°C per cycle) to allow the formation of heteroduplexes in case a mutation is present in the pool. *LCYE* fragment 1 was digested with celery juice extract (CJE) extracted from celery following the protocol described by Till et al., 2006 [40]. CJE contains an endonuclease (CEL1 mismatch endonuclease from *Apium graveolens* [celery]) that identifies the mismatched pair of bases in the heteroduplex and cleaves fragment 1 into two fragments. A test with a known gene mutant was performed to confirm CEL1 activity and optimal amount of CJE for heteroduplex digestion (S2 Appendix). Digestion was performed with 15 μL PCR product, 2 μL CJE, 1,7 μL 10x digestion buffer [40], and 1.3 μL dH_2_O. The digestion was carried out at 45°C for 45 min and stopped immediately by adding 5 μL of 0.225 M EDTA per sample, and then vortexed thoroughly.

Digested PCR products were separated by vertical electrophoresis in 6% polyacrylamide gels (19:1 acrylamide:bis ratio) in 0.5X TBE running buffer at 1,600 V for 45–60 min, and stained with silver nitrate. Samples were also visualized on a 2% agarose gel in 1X TAE running buffer, and subsequently visualized using GelRed DNA gel stain (Biotium Inc.) under UV light. Both gel images were analyzed and mutants pools were identified by the presence of cleaved products whose combined size was similar to fragment 1 size (~1,700 bp).

### Identification of mutant individuals

The second screen was performed using 2X pools of individual DNAs from the mutant 6X pools from the first screen, with each DNA being present in two 2X pools. Each individual named as A, B, C, D, E, F was part of the mutant 6X pool and they were assembled in pairs (e.g. A+B, B+C, C+D, D+E, E+F, F+A, 10 μL from each) to look for *LCYE* mutants. This step followed the same detection protocol as described above. *LCYE* mutant individuals were identified as they showed the presence of cleaved products in both of the 2X pools where they were present.

### Sequencing analyses

Four non-mutant individuals of each genome plus eight *LCYE-A* and eight *LCYE-B* putative mutants were used (S3 Appendix).

Sequences were analyzed with Geneious 4.8.3 Software (Biomatters Ltd, 2009). First, an assembly was made with fragments 1a and 1b; forward and reverse sequences of the 3 repeats for all mutant and control sequences were used. Each mutant consensus contig was aligned with the control consensus contig of the respective genome and mutations were identified. The nucleotide sequence was aligned with the *LCYE* hexaploid variety Chinese Spring sequences (EU649785 for *LCYE-A* and EU649786 for *LCYE-B*) for a proper translation and mutant aminoacids identification.

Substitutions in mutants were analyzed with SIFT (Sorting Intolerant From Tolerant), BLOSUM62 and PSSM (Position-Specific Scoring Matrix) algorithms [41]. PSSM positive scores indicate that the given aminoacid substitution occurs more frequently in the alignment than expected by chance at a given position, while negative scores indicates that the substitution occurs less frequently than expected. In contrast, BLOSUM62 matrices are position-independent matrices, in which a residue substitution receives the same score regardless of the position. Residues with positive BLOSUM62 scores substitute frequently in homologous proteins, whereas those with negative scores are rarely substituted [42]. SIFT predicts whether an aminoacid change affects protein function based on the degree of conservation of aminoacid residues in sequence alignments. In this way, negative BLOSUM62, positive ΔPSSM (PPSM control—PSSM mutant) values (>10) and low SIFT scores (<0.05) [41] predict mutations that significantly affect protein function and/or may produce a knockdown or knockout mutation.

### Phenotyping

#### Plant material

Ten seeds per mutant line progeny with affected protein function were provided by the Dubcovsky laboratory from the University of California in Davis, United States. Seeds were grown in a greenhouse with controlled conditions. DNA was extracted using the CTAB method [43]. Specific primers were designed to differentiate mutant from non mutant individuals of the segregant progeny (Table 2). Grains from mutant *LCYE-A* and *LCYE-B* plants were harvested at physiological maturity (12%-14% seed moisture content). Grains were stored at 4°C until extraction.

**Table 2:** Primer sequences (forward and reverse) used to identify the mutant and non mutant (control) individuals from each progeny line.

LINE	SPECIFICITY	FORWARD	REVERSE	TM
2426	MUTANT	TGTCTGATATTTCAGCATGA	TCGCAGGCTAGTCAAATGAA	60
	CONTROL	TGTCTGATATTTCAGCATGG		
610	MUTANT	AGATCTTTGTCTGAAGCTCT	TCTCGCAGACTAGTCATCAA	62
	CONTROL	AGATCTTTGTCTGAAGCTCC		
457	MUTANT	CTGAGAAGGTACATTCGATC	ACTTTTATGATACGAACTCT	59
	CONTROL		ACTTTTATGATACGAACTCC	
2674	MUTANT	CTGAGAAGGTACATTCGATC	TGAGGATTTGTATGTACCAA	58
	CONTROL		TGAGGATTTGTATGTACCAG	

#### Pigment extraction from leaves

To extract pigments from leaves, 300 mg of tissue was collected and macerated in liquid nitrogen in sterilized morter. 8 mL of hexane:acetone:ethanol solution (2:1:1) were added to each sample and then homogenized. Then, the mix was transferred to a 15 mL tube and vortexed thoroughly for 2 min. Subsequently, each sample was centrifuged at 10,000 g for 10 min at 4°C, and carotenoids were recovered from the upper phase and transferred to a 2 mL tube. During the whole process, the extract was maintained in ice and darkness to avoid carotenoid degradation. Finally, samples were dried using a speedvac device at room temperature and stored at -80°C until quantification and HPLC analysis.

#### Pigment extraction from grains

Whole grains were ground and sieved into fine powder (particles size 250 μm). Carotenoids were extracted using modifications of Howitt et al,. 2009 [29] and Pogson et al., 1996 [18] protocols. 500 mg of flour were mixed in a 15 mL tube with 1 mL of acetone:etil acetate 3:2 (v/v) and vortexed for 2 min, then 1 mL of distilled water was added and then centrifuged for 10 minutes at 13,000 rpm at 4°C. The upper phase was recovered into a 2 mL tube. The process was repeated twice to extract all the pigments. During the whole process, the extract was maintained in ice and darkness as in the section above.

Two technical repetitions were used for A10 and B1, and three biological repetitions for B3 because no enough material to make technical repetitions was available, and the standard deviations between biological repetitions were low.

#### Total carotenoid quantification and High Performance Liquid Cromatography (HPLC) analysis

To quantify the total carotenoid concentration in the leaves pigment extracts from the selected mutants, pigments were resuspended in 1 mL of acetone for Spectrom HPLC analysis. To measure absorbance, the sample was diluted 50 times in acetone and measured in a Shmimadzu spectrophotometer at 750, 662, 645 and 470 nm in quartz cuvettes. To quantify the total carotenoid concentration in grains, pigments were resuspended in 400 μL of acetone for Spectrom HPLC analysis and then diluted 10 times to measure absorbance. Absorbance at 662, 645 and 470 nm allows the determination of chlorophyll *a*, *b* and total carotenoids, respectively, according to Eqs (1), (2) and (3). Absorbance at 750 nm allows the determination of sample turbidity, which is zero when sample is totally transparent, because chlorophyll *a*, *b* and carotenoids did not absorb in this region. Total carotenoid concentrations were calculated using equations outlined in Lichtenthaler and Buschman, 2001 [44], and expressed in μg/mg dry weight.

$$Ca\left(\frac{\mu g}{mL}\right)=11.24\times A_{662}-2.04\times A_{645}$$ (1)

$$Cb\left(\frac{\mu g}{mL}\right)=20.13\times A_{645}-4.19\times A_{662}$$ (2)

Ct(μgmL)=(1000×A470−1.9×Ca−63.14×Cb)214 (3)

To determine leaves and grains carotenoid composition, pigments were separated by a Shimadzu HPLC equipment (LC-10AT) with a diode array using a RP-18 Lichrocart125-4 reverse phase column (Merck), using a mix of acetonitrile:methanol:isopropanol (85∶10:5 v/v) as a mobile phase with 1 mL/min flow rate for 60 min at room temperature in isocratic conditions. Data analysis was performed with the LCsolutions software program. Concentration of each carotenoid was determined with the following equation:

$$Cx=\frac{Ct\times Ax}{At}$$ (4)

Cx: Pigment concentration; Ct: Total carotenoid concentration; Ax: Area below pigment peak; At: Total carotenoid peak area [45]. The carotenoids were identified according to their absorption spectra, retention time and comparison with specific pigment standards, which was corroborated by comparison with the Carotenoids Handbook [46, 47].

## Results

### Identification and description of mutants

There is now a publicly-available TILLING database (wheat-tilling.com) that allowed to identify 76 *LCYE-A* and 128 *LCYE-B* putative mutants. The results reported below are based on the manual screening of the *LCYE* mutants, which showed coincidences with the publicly-available TILLING database, such as mutants 2426 (*LCYE-A*), and mutants 983 and 2674 (*LCYE-B*).

**Fig 2:**
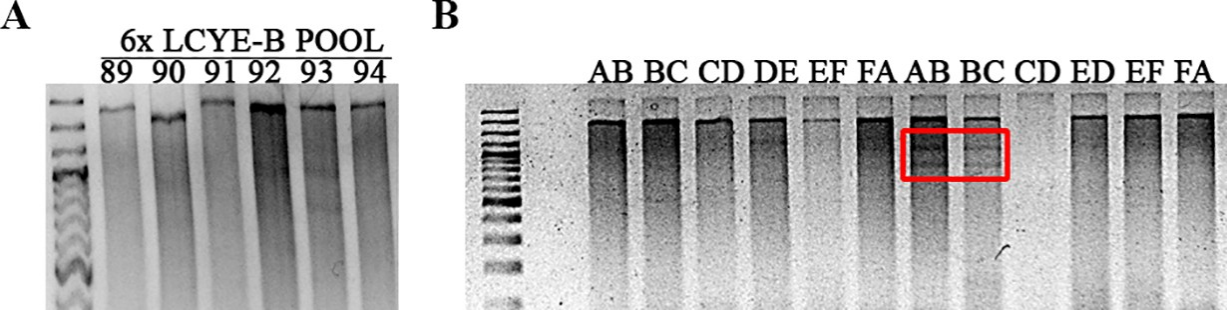
Example of the TILLING platform using mutant line 983. (A) Visualization of 6X pool 93 digested with CJE in a 2% agarose gel. Point mutations in the pools were identified by the presence of two bands whose sizes added up to the full length PCR product. (B) Visualization of paired pools 6X pool 93 individuals (A to F) digested with CJE in a 2% agarose gel. Mutant individual B, which corresponded to line 983 of the Kronos TILLING population, showed the presence of cleaved products in both of the paired pools when present (pool A+B and pool B+C, marked with a red box).

To simplify the TILLING procedure, 6X and 2X pools were made to identify *LCYE* mutants in a more efficient and effective manner. These mutants were identified as they showed CEL1 cleaved products, whose combined size was similar to fragment 1 size (1,764 bp). Twenty seven 6X pools from *LCYE-A* fragment 1 and twenty three pools from the *LCYE-B* fragment 1 were identified as mutants. For example, the sequence of the mutant line 983 for *LCYE-B* is shown in Fig 2. This procedure was repeated for all the selected mutants. In Fig 2A, PCR fragments of 6X pool 93 are visualized after CEL1 digestion where only line 983 showed the expected doble banding pattern.

In the second screen, the individuals from 6X mutant pools were visualized in 2X pools. The individual within 6X mutant pools that contained the point mutation was identified due to double banding pattern visualization. Mutant individual B, which corresponded to line 983 of the Kronos TILLING population, showed the presence of cleaved products in both of the 2X pools when present (pool A+B and pool B+C), which indicates that it corresponds to a mutant line. Then, the PCR product was purified and sequenced. When the sequence was analyzed and compared with the LCYE-B (EU649786) protein reference sequence, it showed a change of G to A, which produces an aminoacidic change from a Valine to an Inuline that may affect the secondary structure of the protein.

Thus, 21 *LCYE-A* and 18 *LCYE-B* possible mutant lines were identified following the protocol previously described (Fig 2B). Fragment 1a and 1b PCR products from these possible mutants lines were then sequenced, and 7 mutations for *LCYE-A* and 11 mutations for *LCYE-B* were identified.

After sequencing, only three mutant lines for both *LCYE-A* and *LCYE-B* were predicted to affect protein function (Table 3, in bold). Mutations in the other mutant candidates do not affect protein function; six mutations at intronic regions that did not produce any change in splice junctions sites, four silence mutations, and two mutations predicted to be tolerated with positive BLOSUM62 scores and high SIFT scores (0.74–0.92) (Table 3).

**Table 3:** *LCYE* mutations identified by sequencing and evaluated by PSSM, BLOSUM62 and SIFT scores.

Gene	Line	Nucleotide change	Amino Acid change	SIFT	ΔPSSM	BLOSUM62	Result
*LCYE-A*	**342**	G3274A	G368R	0.0	9	-2	AFFECT PROTEIN FUNCTION
	1213	G3300R	K376K				SILENCE MUTUATION
	**2426**	G3684A	W437*				EARLY STOP CODON
	**2675**	C3095T	P334L	0.01	10	-3	AFFECT PROTEIN FUNCTION
	2719	G3564A	INTRON				TOLERATED
	593	C3536T	P432S	0.74	0	-1	TOLERATED
	2255	C3380T	INTRON				TOLERATED
*LCYE-B*	**457**	C2916T	G352R	0.01	9	-2	AFFECT PROTEIN FUNCTION
	**610**	C3237T	P405L	0.01	12	-3	AFFECT PROTEIN FUNCTION
	612	G2942A	E360E				SILENCE MUTUATION
	**983**	C2934T	V358I	0.92	0	2	TOLERATED
	217	G3633A	INTRON				TOLERATED
	2283	G3371A	INTRON				TOLERATED
	2231	G1452A	INTRON				TOLERATED
	2324	C2846T	C328C				SILENCE MUTUATION
	**2674**	C3118T	T393I	0.01	5	-1	AFFECT PROTEIN FUNCTION
	2709	C1012T	INTRON				TOLERATED
	638	G2671A	E504E				SILENCE MUTUATION

* PSSM and SIFT scores are not reported for mutations that cause premature stop codons, neither for mutations in introns. In the nucleotide/aminoacid change column, the first letter indicates the wild type nucleotide/amino acid, and the number is the position of the reference gene (*LCYE-A* Genbank: EU649785 and *LCYE-B* Genbank: EU649786) from the start codon/methionine. The last letter corresponds to the mutant nucleotide/aminoacid. Bold mutant lines indicates the most disruptive changes in protein function.

### Mutant identification in progeny

**Fig 3:**
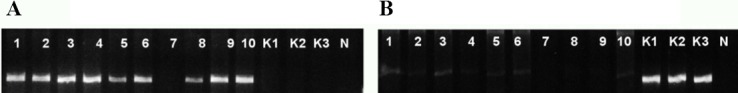
Amplification of Fragment 1 from seeds of line 2674 *LCYE-B* mutant. Amplification of Mutant-specific markers (A) and Control-specific Markers (B). Numbers 1 through 10 indicate the different seeds from line 2674 progeny, and K1, K2, K3 are wild type Kronos used as control.

Ten seeds from each mutant identified by sequencing (Table 3) were sown and mutations were checked by amplification of fragment 1 (Fig 3). In order to obtain a final null phenotype, the selection of sister lines in homozygous state is an efficient strategy. 3 *LCYE* mutant plants from line 2426; 1 from line 610 and 9 from line 2674 were obtained. No mutants were obtained from line 457 (Table 4). The reduction of mutants between Tables 3 and 4 are due to the letality of lines 342, 2675, and line 457 did not generate progeny.

**Table 4:** Number of *LCYE-A* and *LCYE-B* mutants identified (specific primers in Table 2), with their corresponding aminoacid change, and their crossing generation.

Gene	LINE	Mutant Name	Generetion of crossing	Amino Acid change	N° of mutants identified
*LCYE-A*	2426	A1	M4	W437	3
*LCYE-B*	610	B1	M5	P405L	1
	457	B2	M3	G352R	0
	2674	B3	M4	T393I	9

### Carotenoid quantification in leaves and grains of mutants

**Fig 4:**
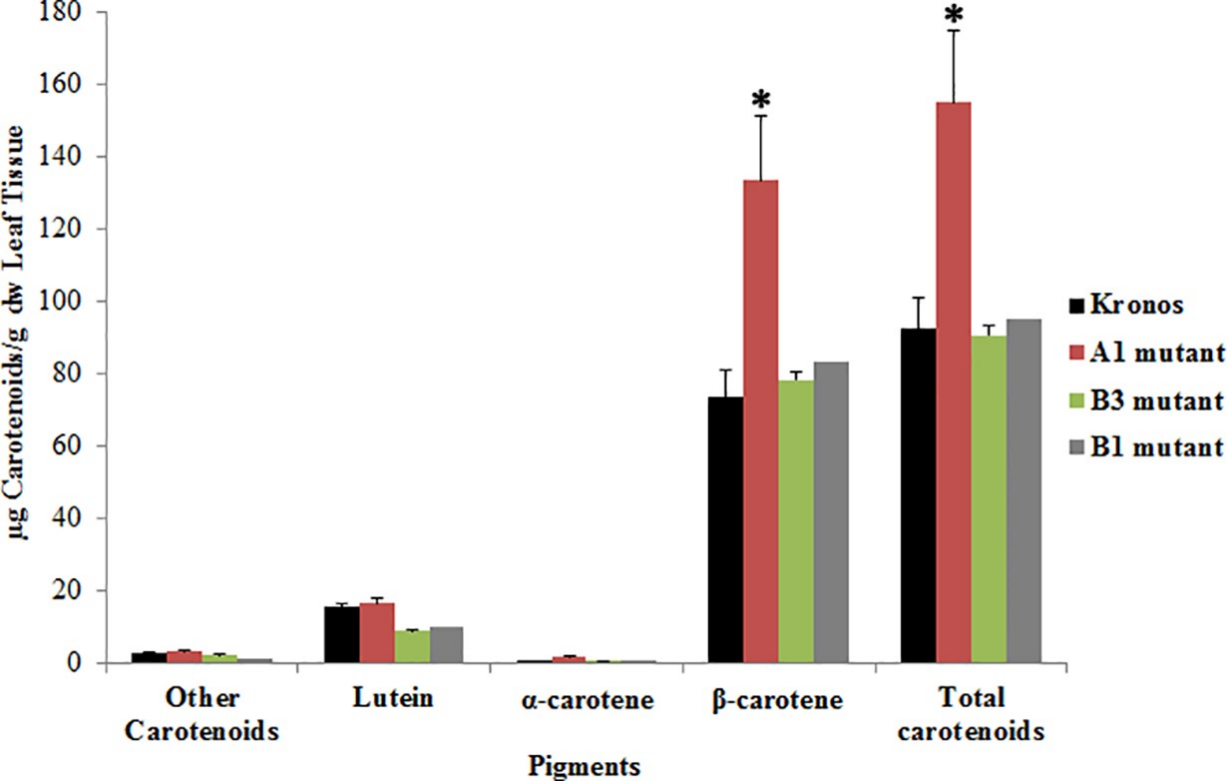
Carotenoid content in leaves of the selected *LYCE* mutants. Total carotenoid content (carotenes and xanthophylls), β-carotene, lutein and α-carotene are shown. The values obtained for each pigment correspond to samples comprising three biological replicas except for B1 mutant in which only one mutant plant was studied. Two-way ANOVA using the GraphPad Prism 5 software was performed to determine significant differences between samples, *: p<0.05.

**Fig 5:**
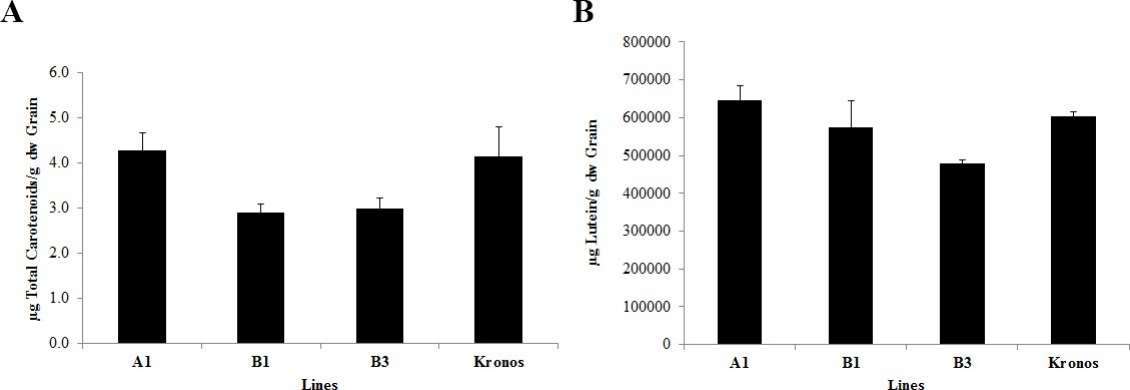
Total carotenoid content in grains of the selected *LCYE* mutants. (A) Total carotenoid levels were measured at 470 nm for A1, B1 and B3 mutants and correspond to three biological replicates. Non-paired two tails Student t-test was performed to determine significant differences between samples, and no statistical differences were detected between the lines. (B) Lutein content in grains of the selected *LCYE* mutants. Lutein levels were measured by HPLC for A1, B1 and B3 mutants and correspond to three biological replicas. Non-paired two tails Student t-test was performed to determine significant differences between samples, and no statistical differences were detected between the lines.

The analysis of total carotenoids in leaves showed that the mutant A1 from line 2426 had a significant β-carotene increase relative to the control plants, which associated to an increase in 75% of β-carotene compared to Kronos, with no change in lutein content (Fig 4, S2 Fig). Although the *LCYE-B* mutants had a functional mutation in the gene, no difference in the amount of the total and the specific carotenoids was observed (Fig 4). Unexpectedly, no significant difference in total carotenoids (Fig 5A) nor lutein (Fig 5B) was observed in grains between the A1, B1 and B3 mutants compared to Kronos, although the amount of pigments was lower in the *LCYE-B* mutants.

## Discussion

### Identification of mutants by screening

*LCYE* mutants were identified following a two-step screening approach, outlined by Uauy et al., 2009 [32]. First, 27 pools from *LCYE-A* fragment 1 and 23 pools from *LCYE-B* fragment 1 were identified. In the second screen, individuals within 6X pools that contained point mutations were found. Depending upon the banding pattern, the mutation was assigned to one of the six individual DNAs; 21 *LCYE-A* and 18 *LCYE-B* possible mutant lines were identified. The fact that more mutants were found in the first screen could depend on several factors, such as the presence of non-specific PCR products due to false heteroduplexes. Furthermore, the efficiency of heteroduplex formation is directly proportional to the pool size [48, 49], and 6X pools are accordingly more efficient than 2X pools. PCR quality and quantity are important for agarose gel detection, and the sensitivity on agarose gels is low: products with low concentrations (i.e., 5 ng/μL) are normally not detected on regular agarose gels [50]. Consequently, if some lower bands (corresponding to the digested fragments) were not visible, it could be possible that PCR was not concentrated enough. However, an advantage of the agarose detection method is that it has lower cost and it is easier to manage than acrylamide gels. Besides, agarose gels have been used successfully in other wheat TILLING studies [35, 50–53].

### Sequencing analysis

Fragment 1a and 1b PCR products from the possible mutant lines were sequenced; 7 mutations for *LCYE-A* and 11 mutations for *LCYE-B* were identified. Lines that produced fragments smaller than 200 bp when digested by CJE could not be detected by sequencing. Mutations density in a tetraploid wheat population is approximately 1/51 kb for 50% GC targets [32]. This is corrected for 50% GC content because it depends on the GC content of the target regions used, since the EMS mutagen acts on GC residues. *LCYE-A* and *LCYE-B* fragment 1 contain approximately 41% GC content, thus lower mutation density is expected. Other factors that explain differences in mutation densities are the different EMS doses, different genotypes and the detection method used [51]. Sequencing analysis confirmed that 100% of the mutations were G to A or C to T transitions, as expected from alkylation by EMS [31].

One of the disadvantages of using CEL1 based TILLING is that it is less productive to screen genes with multiple small exons separated by larger introns, because mutations in introns rarely affect gene function [54]. Fragment 1 has 7 exons that occupied 54% of the region, consequently nearly half of the fragment 1 was not useful to identify important mutations. Six of the eighteen mutations found were intronic mutations that did not affect gene function.

### Phenotypic analyses of the identified LCYE mutations

Lines 342, 2675, 457 and 610 contained mutations in LCYE-A G368R, P334L and LCYE-B G352R, P405L, respectively (Table 3). These mutations exhibited similar SIFT scores and these changes were predicted to affect protein function. P405L and P334L had more significant scores, PSSM score is (>10) and BLOSUM is (-3), but no phenotypic analysis could be carried out since lines 342 and 2675 were lethal and line 457 had no mutant progeny. One of the problems with this technique is that TILLING mutant populations are mantained without selection, therefore mutations can be lost after a few generations of crossing, especially if they decrease plant fitness.

Phenotypic analysis was difficult to be achieved since plant material was scarse. Furthermore, an interesting variation in β-carotene and total carotenoid content could be observed by the generation of null mutant lines as a result of crossing A and B *LCYE* mutant lines, and by selecting homozygous double mutant lines in the F_2_ generation. Line 2426 (mutant A1, Tables 3 and 4) showed a signicant β-carotene and total carotenoid increase in leaves based on the HPLC analyses (Fig 4, S2 Fig). This result is very valuable for plants produced under stressful environmental conditions, with high concentrations of polycyclic aromatic hydrocarbons (PAHs), which are volatiles compounds that can cause severe toxicity in plants [55]. Using a multi-faceted approach, Shen et al., 2018 [56] reported that the carotenoid content in leaves and superoxide dismutase are the two most effective antioxidants involved in ROS scavenging in the accumulation of phenanthrene in wheat leaves, which have important implications to enhance plant resistence under PAH pollution in the environment.

Line 2426 showed the most promising mutation, W437* in *LCYE-A*, as this substitution resulted in a premature stop codon. This mutation eliminated exons 9 and 10, which encoded for the last 94 amino acids of the protein. This protein region contains a conserved domain that corresponds to a short-chain Dehydrogenases/Reductases (SDR) domain (Genbank accession: cl21454). This is a functionally diverse family of oxidoreductases that has a single domain with a structurally conserved Rossmann fold (alpha/beta folding pattern with a central beta-sheet), a NAD(P)(H)-binding region, and a structurally diverse C-terminal region [57]. The potential disruptive effect of this mutation was also supported by a high PSSM score (13) of the tryptophan residue (W) in this position. Large positive scores (>10) often indicate critical functional residues, which may be active site residues or residues required for other intermolecular interactions.

No substantial differences in total carotenoid and lutein content were identified and no other specific carotenoids were found in grains (S3 Fig), although mutations obtained in *LCYE-A* and *LCYE-B* predicted to affect protein function. These results can be explained by the fact that LCYE-B could compensate in grains but not in leaves the W437* substitution in LCYE-A offset by the B genome. A similar explanation can be proposed for the *LCYE-B* mutants (B1 and B3 mutants, Figs 4 and 5B), in which LCYE-A may compensate the LCYE-B deficiency. Although in many plants like *Solanum lycopersicum* (tomato), paralog enzymes have organ specific functions [58, 59], others species show that paralogs participate in carotenoid synthesis in the whole plant. This is the case of carrot, in which *LCYB1* silencing produces a redution of carotenoids in leaves and in a minor degree in the storage roots, probably as a compensation mechanism carried out by the paralog *LCYB2* [23]. Qin et al., 2016 [20] showed the expression of both *LCYE* homoeologous across vegetative and reproductive tissue in the Kronos durum wheat cultivar. Their findings reveal that both the A and B genome copies exhibit increasing levels of LCYE expression in grains, as opposed to lutein concentration, thus complex mechanisms on lutein accumulation are involved. In vegetative tissues, the expression patterns of both genes are consistent with the light harvesting and the photoprotective role of carotenoids in photosynthetic tissues. Furthermore, as both genes are expressed in the tested tissues, a compensatory response in the absence of one or the other *LCYE* gene likely occurs. Therefore, expression analysis and functional characterizations of the *LCYE* genes in durum wheat have to be carried out to prove this compensation hypothesis.

### Differences between Kronos and Chinese Spring varieties

The sequences were aligned with the reference gene and some differences between varieties were observed. Two SNPs were identified between these varieties for *LCYE-B*, one in an intron and W484S, which predicted to affect protein function by low SIFT score (0.04) and a high PSSM score (15) (Table 5). Tryptophan is predicted as a critical functional residue at 484 position [41], and protein function is likely affected when this residue is substituted.

**Table 5:** Differences of *LCYE-B* sequences between Kronos and Chinese Spring varieties.

Gene	Nucleotide change	Amino Acid change	SIFT	Δ PSSM	Result
*LCYE-B*	C3766G	W484S	0.04	15	AFFECT PROTEIN FUNCTION
	A3656G	INTRON			

The SNP identified in *LCYE-B* (corresponding to W484S substitution) using the Kronos TILLING population was also detected in the Sunco x Tasman doubled haploid population, developed by Kammholz et al., 2001 [60], at the start of exon 10 of the *LCYE-B* homeologues [29]. Tryptophan (W) in the substitution W484S is conserved between a range of LCYB and LCYE protein sequences [61]. Aminoacid alignments revealed that this tryptophan is conserved in more than 20 plant, algal and cyanobacteria species, except for two LCYEs from *Gentiana lutea* (Genbank: BAA88845) and *Coffea canephora* (Genbank: ABC87738) [29]. In a multialignment of 100 protein sequences of different species obtained by BLAST analysis of LCYE-B, 94 sequences showed a tryptophan in this position and 5 of them a serine; *G*. *lutea* and *C*. *canephora* as mentioned previously, but also a substitution in *Sesamum indicum* chloroplastic LCYE (Genbank: XP011078026), *Genlisea aurea* (Genbank: EPS63769), and *Erythranthe guttata* (GenBank: EYU28375.1) hypothetical proteins. *G*. *lutea* LCYE exhibited enzymatic activity [62], and *C*. *canephora* contained low β-carotene levels and high levels of α-carotene in leaves [63], which is atypical in leaf tissue [64]. This result indicates that the serine substitution is associated with higher endosperm lutein content and potentially an increase in LCYE activity relative to LCYB, explaining the increased α-carotene and lutein contents in wheat and other species. Further studies need to be conducted to confirm this result.

### Future directions and conclusions

The mutants identified in this study need to be backcrossed with wild type Kronos, and then selected for several generations to introgress the *LCYE* mutations, which will reduce the background mutations that can negatively affect the general performance of the future variety. The TILLING technique was used in this study to identify point mutations in a population mutagenized by EMS of 1,370 individuals of Kronos durum wheat. 21 mutants for the *LCYE-A* gene and 18 for the *LCYE-B* gene were identified. Through the analysis of their sequences, point mutations were identified in the *LCYE* genes that were associated to important substitutions in the LCYE proteins. Substitutions W437*, P334L, G368R in LCYE-A, and P405L, G352R, T393I in LCYE-B showed a high probability of producing a truncated enzyme that diminished its efficiency or losed its function, according to the PSSM, SIFT and BLOSUM62 algorithms. There is evidence that the alteration or loss of function of the LCYE enzyme can increase the flow of the β-β branch in the biosynthesis of carotenoids, augmenting the content of β-carotene. In our study, substitution W437* increased β-carotene in 75% and overall total carotenoid content in leaves of the mutant 2426 (A1 mutant line), which have potential applications to improving plant resistance under polluted environmental conditions. The TILLING technique allowed our group to find new alleles with a high probability of increasing β-carotene content in durum wheat. These alleles are stably inherited and can be used in traditional breeding programs since mutagenesis is not considered a transgenic technique, meaning that they have no legal restrictions, nor high costs due to licenses or social opposition. These new alleles could also be used in functional genomics to study the individual contribution of *LCYE-A* and *LCYE-B* to the final content of carotenoids, and help to elucidate the functioning of the biosynthetic pathway of carotenoids in wheat.

## Supporting information

S1 Fig Genome-specific primers validation. Langdon D-genome disomic substitutions lines LDN-3D(3A) (the genome B is present) and LDN-3D(3B) (the genome A is present) in which the A and B genome are substituted by the D genome, respectively, were used to validate the genome specificity of the designed primers. PCR was carried out using the genome specific primers highlighted in grey, to amplify DNA from LDN-3D(3A) and LDN-3D(3B) lines (3A and 3B in figure), and water as a negative control (N). Genome specific primers should not be able to amplify the substitution line for the respective genome, for example *LCYE-A* genome specific primers showed no PCR product when LDN-3D(3A) DNA was used. These pictures validate that primers LCYE-BF1, LCYE-BR1 (a), LCYE-AF1, LCYE-AR1 (b) and LCYE-BF2 and LCYE-BR2 (c) were genome specific. LCYE-BF2 and LCYE-BR2 (d) were not genome specific as they amplified fragments for both genomes.
(DOCX)

S2 Fig Chromatogram of leaf tissue showing the carotenoids composition. Peak a corresponds to lutein, peak c to chlorophyll *a*, peak d to α-carotene and peak e to β-carotene. Chromatogram was obtained through HPLC pigment analysis and compares mutant A1 and Kronos leaf samples.
(DOCX)

S3 Fig Chromatogram of grains showing the carotenoids composition. Only lutein showed a peak in the chromatogram through HPLC pigment analysis. A representative chromatogram of mutant A1 is reported in this figure.
(DOCX)

S1 Table LCYE fragments amplified by PCR with their respective primer acronym, size (bp) and melting temperature TM (°C). (DOCX)

S1 Appendix Touchdown PCR conditions. (DOCX)

S2 Appendix CEL1 calibration. (DOCX)

S3 Appendix PCR for sequencing. (DOCX)
