# Supplementary figures and images for: Identification of Lycopene epsilon cyclase (LCYE) gene mutants to potentially increase β-carotene content in durum wheat (Triticum turgidum L.ssp. durum) through TILLING
Source: PLoS One. 2018 Dec 10;13(12):e0208948. doi: 10.1371/journal.pone.0208948 (PMC6287857; doi:10.1371/journal.pone.0208948)

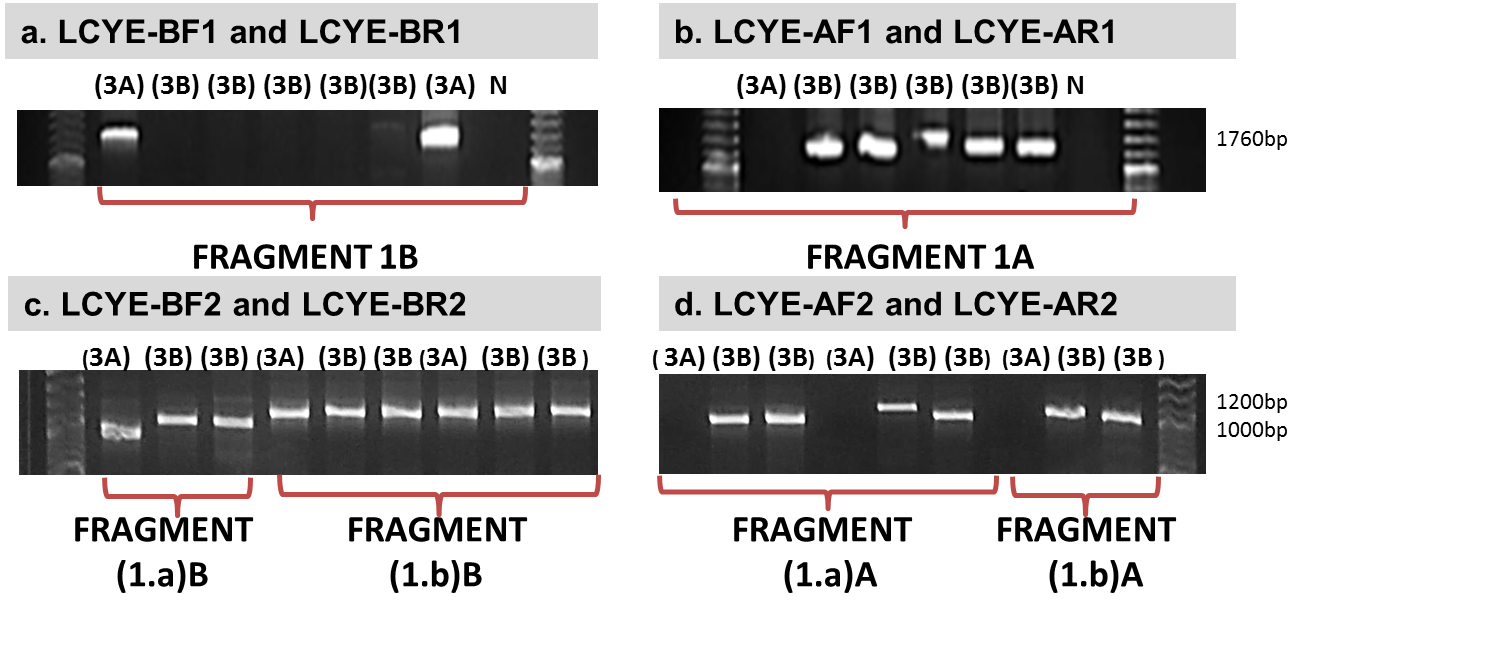

Supplement: S1 Fig — Langdon D-genome disomic substitutions lines LDN-3D(3A) (the genome B is present) and LDN-3D(3B) (the genome A is present) in which the A and B genome are substituted by the D genome, respectively, were used to validate the genome specificity of the designed primers. PCR was carried out using the genome specific primers highlighted in grey, to amplify DNA from LDN-3D(3A) and LDN-3D(3B) lines (3A and 3B in figure), and water as a negative control (N). Genome specific primers should not be able to amplify the substitution line for the respective genome, for example LCYE-A genome specific primers showed no PCR product when LDN-3D(3A) DNA was used. These pictures validate that primers LCYE-BF1, LCYE-BR1 (a), LCYE-AF1, LCYE-AR1 (b) and LCYE-BF2 and LCYE-BR2 (c) were genome specific. LCYE-BF2 and LCYE-BR2 (d) were not genome specific as they amplified fragments for both genomes. (DOCX) [file pone.0208948.s001.docx]

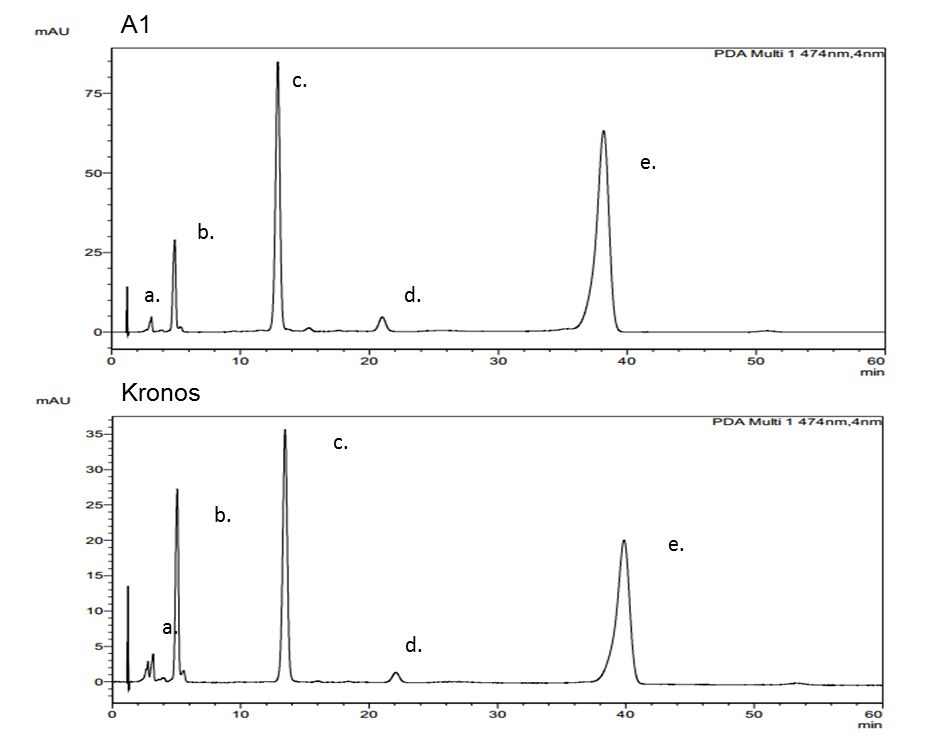

Supplement: S2 Fig — Peak a corresponds to lutein, peak c to chlorophyll a, peak d to α-carotene and peak e to β-carotene. Chromatogram was obtained through HPLC pigment analysis and compares mutant A1 and Kronos leaf samples. (DOCX) [file pone.0208948.s002.docx]

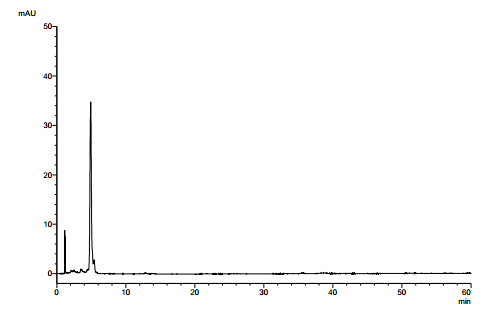

Supplement: S3 Fig — Only lutein showed a peak in the chromatogram through HPLC pigment analysis. A representative chromatogram of mutant A1 is reported in this figure. (DOCX) [file pone.0208948.s003.docx]
