# Supplementary material for: Identification of Lycopene epsilon cyclase (LCYE) gene mutants to potentially increase β-carotene content in durum wheat (Triticum turgidum L.ssp. durum) through TILLING
Source: PLoS One. 2018 Dec 10;13(12):e0208948. doi: 10.1371/journal.pone.0208948 (PMC6287857; doi:10.1371/journal.pone.0208948)
Supplement: S1 Table — (DOCX) [file pone.0208948.s004.docx]

| Fragment | Forward | Reverse | Size (bp) | TM(°C) |
| --- | --- | --- | --- | --- |
| LCYEA fragment 1 | LCYE-AF1 | LCYE-AR1 | 1760 | 50.3 |
| LCYEB fragment 1 | LCYE-BF1 | LCYE-BR1 | 1764 | 55 |
| LCYEA fragment 1.a | LCYE-AF1 | LCYE-AR2 | 1000 | 55 |
| LCYEA fragment 1.b | LCYE-AF2 | LCYE-AR1 | 1200 | 55 |
| LCYEB fragment 1.a | LCYE-BF1 | LCYE-BR2 | 1000 | 58 |
| LCYEB fragment 1.b | LCYE-BF2 | LCYE-BR1 | 1250 | 58 |
