# Supplementary material for: Identification of Lycopene epsilon cyclase (LCYE) gene mutants to potentially increase β-carotene content in durum wheat (Triticum turgidum L.ssp. durum) through TILLING
Source: PLoS One. 2018 Dec 10;13(12):e0208948. doi: 10.1371/journal.pone.0208948 (PMC6287857; doi:10.1371/journal.pone.0208948)
Supplement: S1 Appendix — (DOCX) [file pone.0208948.s005.docx]

**S1 Appendix. Touchdown PCR conditions.** PCR was carried out in a 25 l reaction volume using 50 ng of 6X pooled DNA, 1 unit (U) of Taq DNA polymerase (Thermo Fisher Scientific) and the following cycling conditions: initial denaturation at 95°C for 2 min, followed by 14 cycles of touchdown at 94°C for 20 s, from 57-66°C to 50-58°C for 30 s (0.5°C decrease per cycle) (according to the annealing temperatures of different primers sets, S1 Table), and extension at 72°C for 75 s. These touchdown cycles were followed by 37 cycles of 94°C for 20 s, 50-58°C for 30 s and 72°C for 75 s.
