# Supplementary material for: Identification of Lycopene epsilon cyclase (LCYE) gene mutants to potentially increase β-carotene content in durum wheat (Triticum turgidum L.ssp. durum) through TILLING
Source: PLoS One. 2018 Dec 10;13(12):e0208948. doi: 10.1371/journal.pone.0208948 (PMC6287857; doi:10.1371/journal.pone.0208948)
Supplement: S2 Appendix — (DOCX) [file pone.0208948.s006.docx]

**S2 Appendix. CEL1 calibration.** To confirm CEL1 activity and optimal amount of CJE for heteroduplex digestion, a *CBF* (cold-inducible gene family, 826 bp) known mutant of the Kronos TILLING mutant population was used, and mixed it with a *CBF* non-mutant creating a 2X pool to form the heteroduplex, which was digested into two fragments of 580 and 240 bp.
