# Supplementary material for: Identification of Lycopene epsilon cyclase (LCYE) gene mutants to potentially increase β-carotene content in durum wheat (Triticum turgidum L.ssp. durum) through TILLING
Source: PLoS One. 2018 Dec 10;13(12):e0208948. doi: 10.1371/journal.pone.0208948 (PMC6287857; doi:10.1371/journal.pone.0208948)
Supplement: S3 Appendix — (DOCX) [file pone.0208948.s007.docx]

**S3 Appendix. PCR for sequencing.** A first amplification was carried out with genome-specific primers to form *LCYE-A* and *LCYE-B* fragment 1, and then an in-between amplification using fragment 1 PCR product as template for amplifying fragments 1a and 1b. PCR was performed in a 25 μL reaction volume using 50 ng DNA and 2 μL of PCR product, 1U of Platinum Taq DNA Polymerase (Thermo Fisher Scientific), and the following cycling conditions: initial denaturation at 95°C for 2 min, 37 cycles of 94°C for 20 s, 50.3-58°C (Table 2) for 30 s and 72°C for 75 s, and final extension of 72° for 10 min. Finally, the PCR products of the two overlapping fragments were purified and sequenced by Macrogen Inc. (Seoul, Korea).
